# Supplementary material for: Flexible hyperspectral surface plasmon resonance microscopy
Source: Nat Commun. 2022 Oct 29;13:6475. doi: 10.1038/s41467-022-34196-7 (PMC9617892; doi:10.1038/s41467-022-34196-7)
Supplement: Supplementary file 1 — Supplementary Information [file 41467_2022_34196_MOESM1_ESM.pdf]

Supplementary information

Flexible hyperspectral surface plasmon resonance microscopy

Ziwei Liu [1,2], Jingning Wu [1,2], Chen Cai [1,2], Bo Yang [1,2], and Zhi-mei Qi [1,2,3,]*

[1] State Key Laboratory of Transducer Technology, Aerospace Information Research Institute, Chinese Academy of Sciences, Beijing 100190, China.

[2] School of Electronic, Electrical, and Communication Engineering, University of Chinese Academy of Sciences, Beijing 100049, China.

[3] School of Optoelectronics, University of Chinese Academy of Sciences, Beijing 100049, China.

***Corresponding Author: E-mail: zhimei-qi@mail.ie.ac.cn.**

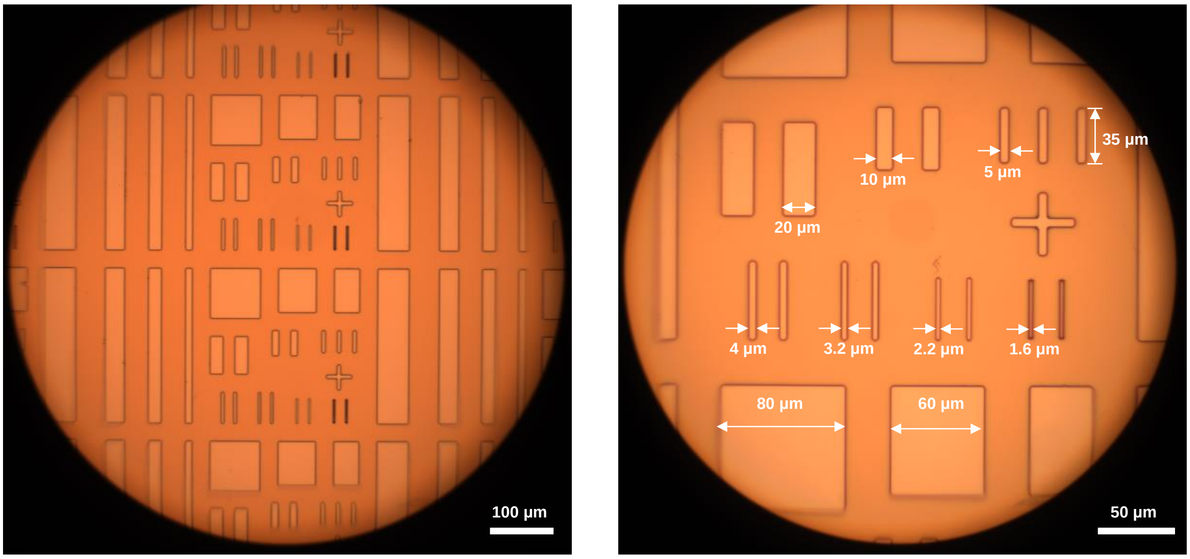

Supplementary Fig. 1 Feature sizes of the patterned SPR chip characterized with an optical microscope (Axio Imager A2, Carl Zeiss Microscopy).

The patterned SPR chip was fabricated by spin-coating a 1.5-μm-thick positive photoresist layer on the gold film followed by standard photolithography, and the patterns marked in the right image is the exposed gold surface.

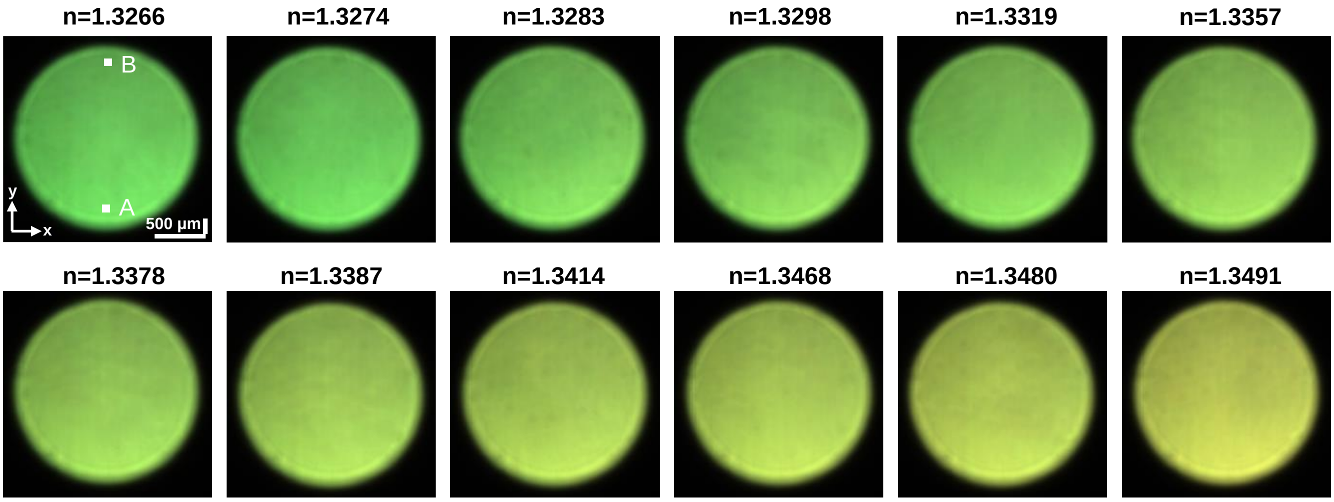

Supplementary Fig. 2 Spectral SPR images of the gold-film SPR chip measured after covering the gold film with aqueous glycerol solutions with different refractive indices ranging from 1.3266 to 1.3491.

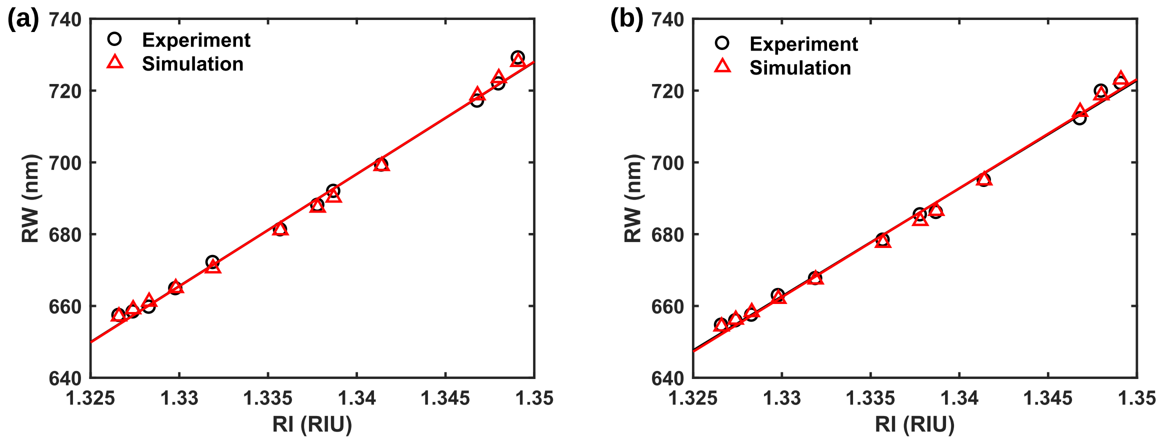

Supplementary Fig. 3 Angular calibration by fitting the experimental single-pixel RI sensitivity using Fresnel formula. (a) Experimental and simulated single-pixel RI sensitivity of pixel A marked in Supplementary Fig. 2, where the experimental sensitivity is 3122 nm/RIU, the gold film thickness calculated to be 44 nm and the incident angle calculated to be 39.80°. **(b)** Experimental and simulated single-pixel RI sensitivity of pixel B marked in Supplementary Fig. 2, where the experimental sensitivity is 3008 nm/RIU, the gold film thickness calculated to be 44 nm and the incident angle calculated to be 40.07°. In the simulation process, we found that the best fit between the simulated and experimental sensitivities could be obtained only when the gold film thickness is 44 nm. Therefore, we assumed that the thickness of the gold film was uniformly distributed at 44 nm, excluding the influence of uneven thickness of gold film on the RI sensitivity.

Supplementary Table 1 TiO_2 film thickness measured by the ellipsometer.

	Point 1	Point 2	Point 3	Point 4	Point 5
Thickness	3.76 nm	3.80 nm	3.85 nm	3.73 nm	3.69 nm
Average			3.77 nm		

We measured thicknesses of five points with spot size of about 1 cm in diameter on the prepared TiO_2 film using an ellipsometer (ESM-300, J.A. Woollam Co.), and the average thickness is 3.77 nm. Using our HSPRM system, the 2D thickness distribution of an area of ~2 mm^2 of the same film was measured to be 3.5±0.1 nm.

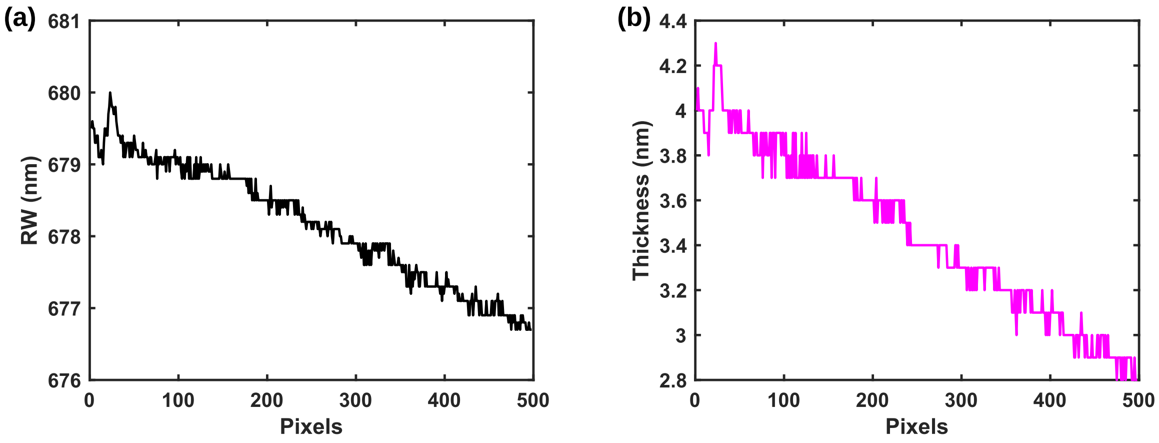

Supplementary Fig. 4 Effect of y-axis incident beam divergence on quantification of BSA adlayer thickness. (a) Simulated RWs obtained with conditions of 44-nm-thick gold film, the calibrated incident angles (39.80°~40.07°) in Fig. 6c, BSA adlayer thickness of 3.5 nm, RI of 1.429 for the BSA adlayer and RI of 1.3325 for the BSA aqueous solution. **(b)** BSA adlayer thickness profile along y-axis calculated with the above simulated RWs and the central calibrated incident angle of 39.94° in Fig. 6c. This simulated result demonstrates that if a BSA adlayer with actual thickness of 3.5 nm measured without angular calibration, the thickness obtained is with a minimum value of 2.8 nm and a maximum value of 4.3 nm. The standard deviation of the BSA adlayer thickness is 0.36 nm, which is 3.6 times as large as that of the 3.5-nm-thick TiO_2 film in Fig. 6, which demonstrates that even a small divergence of the incident angle can seriously affect the quantified results of biochemical samples.

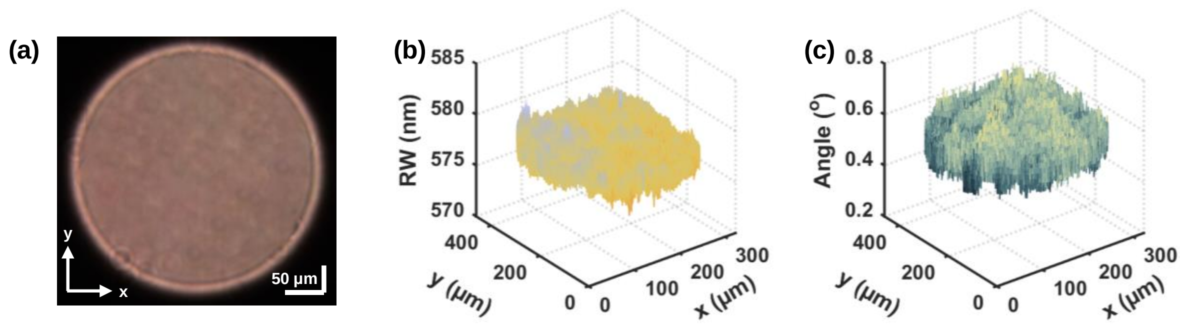

Supplementary Fig. 5 Angular calibration for measuring the thickness of a monolayer graphene. (a) Spectral SPR image of the exposed gold-film area on the SPR ship half-covered with the monolayer graphene. **(b)** 2D RW distribution extracted from the hyperspectral datacube. **(c)** 2D distribution of the actual incident angle obtained by fitting the single-pixel RW using the three-layer Fresnel formula. Compared with the large-area measurement in Fig. 6, the incident angle variation caused by the divergence of incident beam can be neglected in the spatially resolved measurement of small-FOV areas.

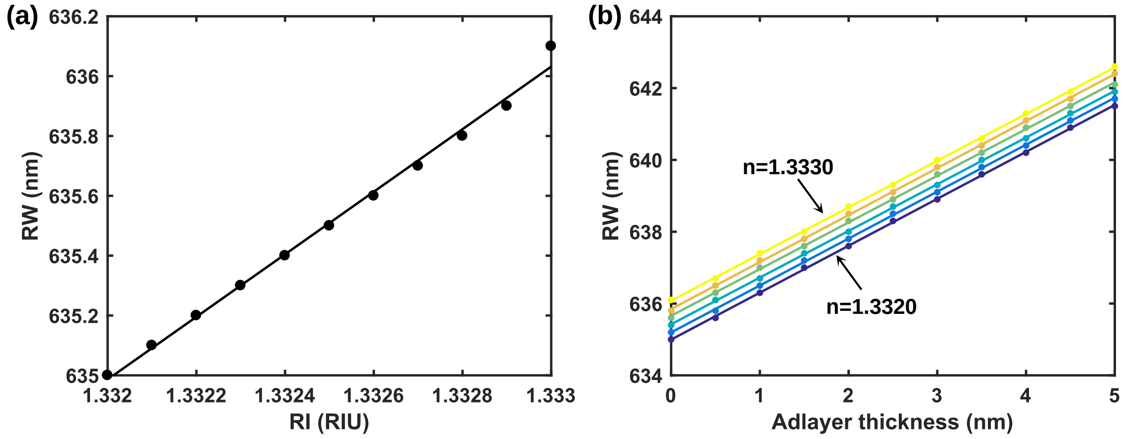

Supplementary Fig. 6 Simulation of SPR sensitivities with respect to bulk RI and protein adlayer thickness. (a) RI sensitivity of the PMMA-film-covered SPR chip. The RW changes by 1.1 nm when the RI of the solution increases by 0.001 RIU. **(b)** Sensitivity of the PMMA-film-covered SPR chip to protein adlayer thickness simulated with different bulk RI ranging from 1.3320 to 1.3330 (the RI interval of 0.0002). The RW varies more than 5 nm when the adlayer thickness increases 5 nm. According to that the relationship between the bulk RI and concentration of the BSA solution is dn/dc=0.188 mL/g[1], the RI difference between the 50 μM BSA aqueous solution and deionized water is calculated to be 6.251×10^{-4} RIU. The variation in RW caused by this small change in bulk RI is negligible compared to that caused by the BSA adlayer thickness. Therefore, it is reasonable that the RW changes measured with the aqueous BSA solution (see Fig. 8f) is attributed to BSA adsotption rather than the bulk RI change.

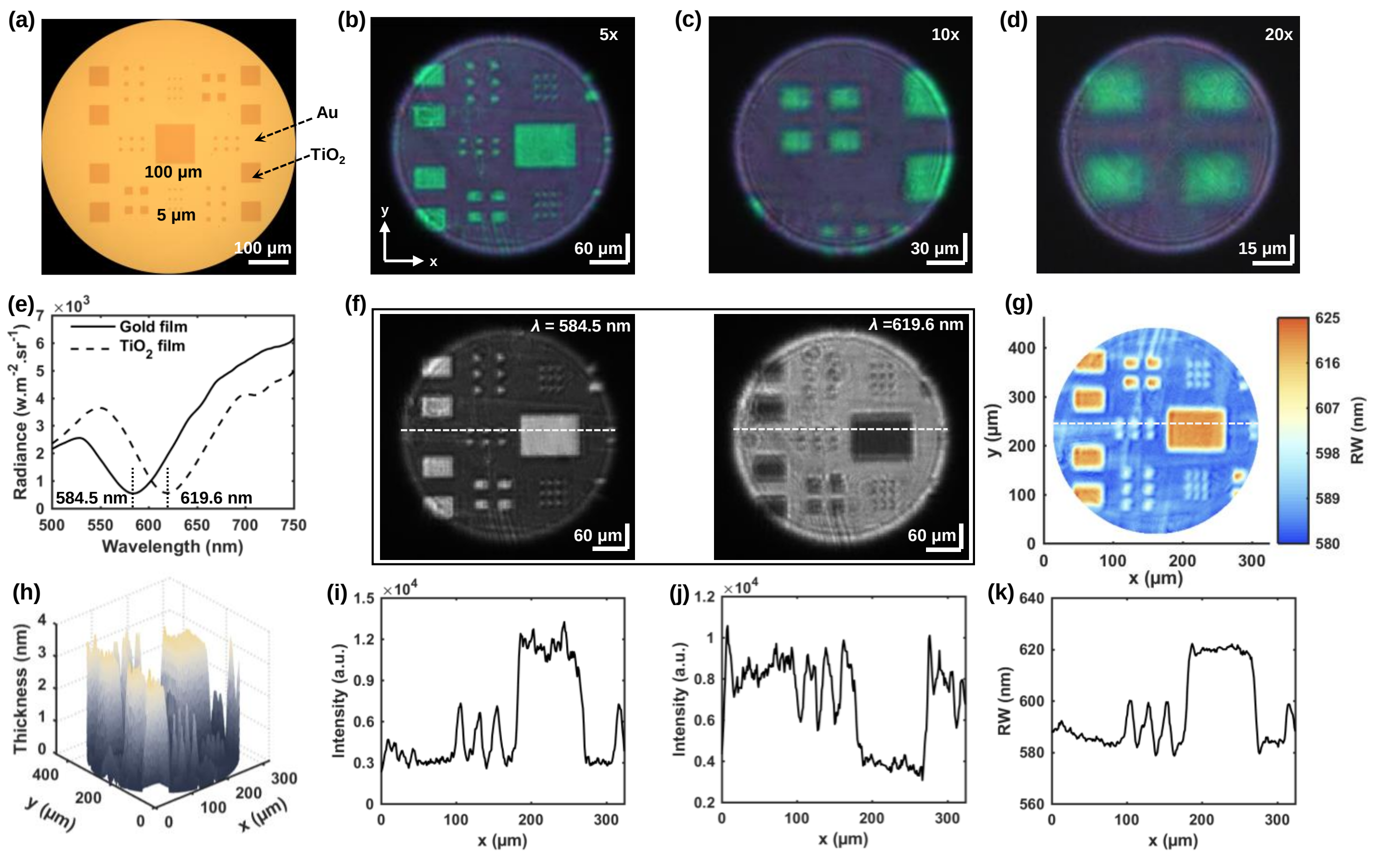

Supplementary Fig. 7 Spectral SPR imaging performance of the HSPRM system with a NA = 0.2 imaging lens fixed at d =2 cm from the prism. (a) RGB image of a patterned ultrathin film of TiO_2 sputtered on the SPR chip observed by a bright-field optical microscope, where the minimum size of the pattern is 5 μm × 5 μm. Spectral SPR images of the same SPR chip measured with **(b)** 5x objective, **(c)** 10x objective and **(d)** 20x objective. **(e)** Single-pixel SPR radiance spectra of the uncovered and TiO_2-film-covered areas of the gold film. **(f)** Greyscale SPR images at λ = 584.5 nm that is the RW of the gold film area (left), and at λ = 619.6 nm that is the RW of the TiO_2-film-covered area of the gold film (right). The contrast at 584.5 nm for the same area of the grayscale image is reversed at 619.6 nm. **(g)** RW image of the SPR chip. **(h)** 2D thickness distribution of the TiO_2 cover layer. **(i)** Single-pixel intensity profile along the dotted line in the left image in **(f)**. **(j)** Single-pixel intensity profile along the dotted line in the right image in **(f)**. **(k)** Single-pixel RW profile along the dotted line shown in **(g)**.

Supplementary Fig. 7 demonstrated that the spectral SPR and greyscale SPR images obtained with NA = 0.2 lens are blurrier than those images obtained with the NA = 0.4 lens (see Fig. 2b and 3b). Although, the RW image has a stronger ability to resist noise than the greyscale SPR image, with the imaging lens L1 (NA=0.2), the RW profile cannot reflect the patten shape and size of the 5-μm patterns. However, as shown in Fig. 3d, with the imaging lens L1 (NA=0.4), the RW profile can reflect the patten shape and size of the 5-μm patterns. Therefore, the spatial resolution of the HSPRM system is mainly determined by the NA of the imaging lens, and higher spatial resolution can be obtained by using a higher NA lens.

Supplementary References

1. Brandenburg, A., Krauter R., Kunzel C., Stefan M. & Schulte H. Interferometric sensor for detection of surface-bound bioreactions. *Appl. Opt.* **39**, 6396-6405 (2000).
