# Supplementary material for: Flexible hyperspectral surface plasmon resonance microscopy
Source: Nat Commun. 2022 Oct 29;13:6475. doi: 10.1038/s41467-022-34196-7 (PMC9617892; doi:10.1038/s41467-022-34196-7)
Supplement: Supplementary file 3 — Reporting Summary [file 41467_2022_34196_MOESM3_ESM.pdf]

nature portfolio

Corresponding author(s): Zhi-mei Qi

Last updated by author(s): Oct 5, 2022

Reporting Summary

Nature Portfolio wishes to improve the reproducibility of the work that we publish. This form provides structure for consistency and transparency in reporting. For further information on Nature Portfolio policies, see our Editorial Policies and the Editorial Policy Checklist.

Statistics

For all statistical analyses, confirm that the following items are present in the figure legend, table legend, main text, or Methods section.

n/a	Confirmed	
☐	☒	The exact sample size (*n*) for each experimental group/condition, given as a discrete number and unit of measurement
☐	☒	A statement on whether measurements were taken from distinct samples or whether the same sample was measured repeatedly
☒	☐	The statistical test(s) used AND whether they are one- or two-sided<br>*Only common tests should be described solely by name; describe more complex techniques in the Methods section.*
☒	☐	A description of all covariates tested
☒	☐	A description of any assumptions or corrections, such as tests of normality and adjustment for multiple comparisons
☐	☒	A full description of the statistical parameters including central tendency (e.g. means) or other basic estimates (e.g. regression coefficient) AND variation (e.g. standard deviation) or associated estimates of uncertainty (e.g. confidence intervals)
☒	☐	For null hypothesis testing, the test statistic (e.g. *F*, *t*, *r*) with confidence intervals, effect sizes, degrees of freedom and *P* value noted<br>*Give P values as exact values whenever suitable.*
☒	☐	For Bayesian analysis, information on the choice of priors and Markov chain Monte Carlo settings
☒	☐	For hierarchical and complex designs, identification of the appropriate level for tests and full reporting of outcomes
☒	☐	Estimates of effect sizes (e.g. Cohen's *d*, Pearson's *r*), indicating how they were calculated

Our web collection on statistics for biologists contains articles on many of the points above.

Software and code

Policy information about availability of computer code

Data collection	All hyperspectral SPR datacubes were collected using SpecView Version 2.9.2.43 (GaiaField Pro-V10E, Dualix Spectral Imaging).
Data analysis	Data analysis was performed using MATLAB R2016b and Python 3.8. Python and MATLAB were used to extract the RW images from the experimental hyperspectral SPR datacubes. MATLAB was used to calculate thickness and RI values according to the Fresnel formula. The code used to analyze the raw data generated in this study are available from the corresponding author upon request.

For manuscripts utilizing custom algorithms or software that are central to the research but not yet described in published literature, software must be made available to editors and reviewers. We strongly encourage code deposition in a community repository (e.g. GitHub). See the Nature Portfolio guidelines for submitting code & software for further information.

Data

Policy information about availability of data

All manuscripts must include a data availability statement. This statement should provide the following information, where applicable:

- Accession codes, unique identifiers, or web links for publicly available datasets
- A description of any restrictions on data availability
- For clinical datasets or third party data, please ensure that the statement adheres to our policy

The raw files generated in this study have been deposited at https://doi.org/10.6084/m9.figshare.21272856.v1. The processed data covered in this paper is collected in a file named Source Data provided with this paper.

Human research participants

Policy information about studies involving human research participants and Sex and Gender in Research.

Reporting on sex and gender	not applicable
Population characteristics	not applicable
Recruitment	not applicable
Ethics oversight	not applicable

Note that full information on the approval of the study protocol must also be provided in the manuscript.

Field-specific reporting

Please select the one below that is the best fit for your research. If you are not sure, read the appropriate sections before making your selection.

☒ Life sciences ☐ Behavioural & social sciences ☐ Ecological, evolutionary & environmental sciences

For a reference copy of the document with all sections, see nature.com/documents/nr-reporting-summary-flat.pdf

Life sciences study design

All studies must disclose on these points even when the disclosure is negative.

Sample size	No statistical methods were used to predetermine sample size. Sample sizes for all experiments are indicated in Figure legends. Each experiment was performed with different samples at least twice for reproducibility.
Data exclusions	No data was excluded.
Replication	All experiments were replicated at least twice. All attempts at replication were successful (size indicated in Figure legend).
Randomization	Randomization is not relevant to the measurement of the patterned SPR chip because the size of the pattern was known before the measurement. The measurement area of the TiO2 film, the RI sensing area on the gold film, and the protein adsorbed area on the PMMA film were randomly selected. Randomization is not relevant to the measurement of monolayer graphene because the interface needs to be chosen to demonstrate the transition from no graphene to with graphene.
Blinding	This study does not contain experiments where blinding would be applicable.

Reporting for specific materials, systems and methods

We require information from authors about some types of materials, experimental systems and methods used in many studies. Here, indicate whether each material, system or method listed is relevant to your study. If you are not sure if a list item applies to your research, read the appropriate section before selecting a response.

Materials & experimental systems

n/a	Involved in the study	
☒	☐	Antibodies
☒	☐	Eukaryotic cell lines
☒	☐	Palaeontology and archaeology
☒	☐	Animals and other organisms
☒	☐	Clinical data
☒	☐	Dual use research of concern

Methods

n/a	Involved in the study	
☒	☐	ChIP-seq
☒	☐	Flow cytometry
☒	☐	MRI-based neuroimaging
